# Evolutionary Analysis of Functional Divergence among Chemokine Receptors, Decoy Receptors, and Viral Receptors

**DOI:** 10.3389/fmicb.2012.00264

**Published:** 2012-07-26

**Authors:** Hiromi Daiyasu, Wataru Nemoto, Hiroyuki Toh

**Affiliations:** ^1^Department of Bioinformatic Engineering, Graduate School of Information Science and Technology, Osaka UniversityOsaka, Japan; ^2^Division of Life Science and Engineering, School of Science and Engineering, Tokyo Denki UniversitySaitama, Japan; ^3^Computational Biology Research Center, Advanced Industrial Science and TechnologyTokyo, Japan

**Keywords:** chemokine receptors, decoy receptors, viral receptors, GPCR, molecular evolution

## Abstract

Chemokine receptors (CKRs) function in the inflammatory response and in vertebrate homeostasis. Decoy and viral receptors are two types of CKR homologs with modified functions from those of the typical CKRs. The decoy receptors are able to bind ligands without signaling. On the other hand, the viral receptors show constitutive signaling without ligands. We examined the sites related to the functional difference. At first, the decoy and viral receptors were each classified into five groups, based on the molecular phylogenetic analysis. A multiple amino acid sequence alignment between each group and the CKRs was then constructed. The difference in the amino acid composition between the group and the CKRs was evaluated as the Kullback–Leibler (KL) information value at each alignment site. The KL information value is considered to reflect the difference in the functional constraints at the site. The sites with the top 5% of KL information values were selected and mapped on the structure of a CKR. The comparisons with decoy receptor groups revealed that the detected sites were biased on the intracellular side. In contrast, the sites detected from the comparisons with viral receptor groups were found on both the extracellular and intracellular sides. More sites were found in the ligand binding pocket in the analyses of the viral receptor groups, as compared to the decoy receptor groups. Some of the detected sites were located in the GPCR motifs. For example, the DRY motif of the decoy receptors was often degraded, although the motif of the viral receptors was basically conserved. The observations for the viral receptor groups suggested that the constraints in the pocket region are loose and that the sites on the intracellular side are different from those for the decoy receptors, which may be related to the constitutive signaling activity of the viral receptors.

## Introduction

The members of the chemokine (CK) family play important roles in regulating cell migration against inflammation, immune surveillance, and oncogenesis in vertebrates (Zlotnik and Yoshie, 2000). The CKs are classified into four subfamilies: CC, CXC, CX3C, and XC, based on the cysteine positions in their motifs (Zlotnik and Yoshie, 2000). CKs exert their activities through binding to their corresponding receptors. Presently, more than 40 CKs and 18 chemokine receptors (CKRs) have been identified in the human genome: 10 CCRs, six CXCRs, one XCR, and one CX3CR (Nomiyama et al., 2011). The CKR homologs are widely distributed among the vertebrate genomes. For example, homologs have even been identified from sea lampreys, which are one of the most primitive vertebrates (Nomiyama et al., 2011). The amino acid sequence identities among the CKRs and the homologs range from 25 to 80%, and the CKRs constitute a subfamily in the class A G protein-coupled receptors (GPCRs). The CKRs have broad ligand specificities (Nomiyama et al., 2011), and each receptor is able to interact with several CKs, and *vice versa*. This binding promiscuity makes it difficult to develop drugs to pinpoint the specific function of each CKR. Among these receptors, only the structure of CXCR4 has been solved by X-ray crystallography (Wu et al., 2010). Like the other GPCRs, this structure is characterized by the seven transmembrane (TM) helices, although T4 lysozyme was inserted within the intracellular loop (ICL) 3 between the TM helices 5 and 6, to stabilize the crystal. The extracellular cavity of CXCR4 is reportedly larger and wide open, as compared to those of other GPCR structures (Wu et al., 2010).

In addition to the traditional CKRs, five non-signaling CKR homologs have been identified in vertebrate genomes: CCRL1 (also known as CCX-CKR), CCRL2, CCBP2 (D6), CXCR7, and DARC (Duffy antigen receptor; Graham, 2009; Leick et al., 2010; Naumann et al., 2010). They are called “decoy” or “silent” receptors, because they are able to bind to several CKs without ligand-induced signaling. Most of them are constitutively internalized with or without ligands, and only the receptors are recycled to the cell membrane. Their functions are considered to regulate inflammatory responses by controlling the volume of free extracellular CKs, through internalization and degradation (Bonecchi et al., 2010). Like the traditional CKRs, these decoy receptors show a broad CK-binding spectrum. CCRL1 interacts with several homeostatic CC-type CKs (Comerford et al., 2006), whereas CCBP2 and DARC interact with inflammatory CKs (Graham, 2009). CXCR7 interacts with the dual-functional CXC-type CKs (Naumann et al., 2010) without activating G proteins (Thelen and Thelen, 2008). CCRL2 is known to be a multifunctional receptor (Yoshimura and Oppenheim, 2011). Like other decoy receptors, it regulates the amount of free CKs. At the same time, it functions as a receptor for an adipokine called chemerin, although the ligand binding does not induce signaling and the receptor is not internalized even after ligand engagement. DARC is the most distantly related to the CKRs among the five decoy receptors, and was originally identified as a malarial parasite receptor (Bonecchi et al., 2010). The receptor also binds to the CC- and CXC-type inflammatory CKs.

Chemokine receptors homologs have been detected in double stranded DNA viruses, such as herpesvirus and poxvirus. These viruses are considered to have gained these proteins by horizontal gene transfer during the course of evolution (Slinger et al., 2011). The viral receptors are constitutively active without ligands, although some of them can bind to CKs. We studied five groups of viral proteins as described below. E1 is derived only from equid herpesvirus 2 of γ-herpesvirinae, which interacts with CCL11 (Camarda et al., 1999). ORF74 is derived from several γ-herpesviruses, and interacts with a broad range of CXC-type CKs (Maussang et al., 2009). The β-herpesviruses also have several CKR homologs. Among them, UL33 is encoded by the genomes of various vertebrate viruses, although its ligands have not been identified (Gruijthuijsen et al., 2002). On the other hand, the US27, US28, and vGPCRs, which share high sequence similarity, have only been identified in the primate β-herpesviruses (Sahagun-Ruiz et al., 2004). US28 is characterized as a receptor for CC-type ligands (Maussang et al., 2009). Several poxviruses, such as capripox virus, deerpox virus, and yatapox virus, also encode CKR homologs in their genomes. The receptors of poxviruses not only share high amino acid sequence similarity to CCR8, but also the CCR8-like CK-binding profile; that is, high affinity to CCL1 (Najarro et al., 2006). These viral receptors are considered to contribute to the escape from and/or the perturbation of the host immune system, and are involved in inflammatory diseases and cancer (Slinger et al., 2011), although the mechanisms of these receptors in viral pathogenesis remain poorly understood.

The CKRs and their homologs have been classified into three functionally different types, from the viewpoints of ligand binding and signaling. The traditional CKRs bind their ligands, which induce signal transduction. The decoy receptors also bind ligands, although the process does not induce signal transduction. In contrast, the viral receptors exhibit signaling activity without ligand binding. The decoy receptors and the viral receptors are considered to have functionally differentiated after their divergence from the traditional CKRs, by gene duplication or horizontal gene transfer. Therefore, the functional differentiation of these three types is expected to have changed the functional constraints at the amino acid sites responsible for the ligand binding and/or signaling. If the sites involved in the functional differentiation can be identified, then the information about the sites would be helpful to understand the mechanisms for the signaling associated with ligand-induced conformational changes. Several different methods have been developed to detect the amino acid sites involved in the functional differentiation of homologous proteins from a multiple sequence alignment, and they are roughly classified into two types. One of them examines the difference in the evolutionary rate at each alignment site among the proteins with different functions (Gu, 1999; Simon et al., 2002), while the other compares the amino acid compositions at each alignment site among the proteins with different functions (Landgraf et al., 1999; Hannenhalli and Russell, 2000). We applied the latter method, the comparison of amino acid compositions, to identify the sites involved in the functional differentiation of CKR homologs. The difference in the amino acid composition at each alignment site between two groups (CKRs and decoy receptors, or CKRs and viral receptors) was calculated as the Kulback–Leibler (KL) information value (Hannenhalli and Russell, 2000; Ichihara et al., 2004). The sites with large KL information values were selected as the candidates for the functional differentiation. The amino acid residues corresponding to the selected sites were mapped on the tertiary structure of CXCR4. The comparison of the CKRs and decoy receptors revealed that the sites with large KL information values were concentrated on the cytosolic side of the CKR structure, with statistical significance. In contrast, there was no such bias in the distribution of the sites with large KL values between the CKRs and viral receptors. Based on the detected sites and the distribution of the corresponding residues on the tertiary structure, the underlying mechanisms for the functional divergence of the CKR homologs will be discussed.

## Materials and Methods

### Amino acid sequence data

The amino acid sequences of the CKRs and their homologs, including decoy receptors and viral receptors, were collected by searching the non-redundant protein sequence database at the NCBI site^1^ with BLAST version 2.2.25 (Altschul et al., 1997). The amino acid sequence of human CXCR4 (GI number of NCBI: 1705894) was used as the query for the BLAST search. The sequence similarity search was also performed against the Ensembl^2^ and elephant shark genome project^3^ genome databases. When several amino acid sequences were almost identical, one of them was selected as the representative. The sequences used in this study are shown in Table 1.

**Table 1:** **The sequences of the CKRs, decoy receptors, and viral receptors**.

CKRs									
**CCR1**									
416802	114586498	332215794	297206879	3023506	85718627	118150798	48675909	283837817	194221405
1705891	10120494	281343586	209863082	84370370	126341640				
**CCR2 AND CCR5**									
1705896	116243032	2851566	2494974	110278904	213391512	48675899	148234591	301754037	75073875
75073881	75072034	75073877	33521616	9502108	33521612	5712983	75073171	5713007	75075056
75074166	75073886	38605083	75074950	3023510	48428812	48427940	75073874	75069418	75074956
3913250	75073879	6831507	75073880	6831506	75069417	6831508	6831510	75073878	6831511
38604970	3023504	6831509	3023503	38604969	75073883	75073884	75073876	75073882	75074954
75074955	75074952	5713069	5713068	75070083	154813802	13431410	114586511	297712573	332266801
296225031	1168965	291393559	301754035	57101676	147901663	303227941	48675907	10719941	2506483
145226674	145423899	126341644	126341394	149632073	149632071	154813804	326922093	224045497	113951665
224045499	327282149	327282151	327282147	148238158					
**CCR3**									
1705892	149632069	126341642	1705893	6831505	281343587	55976357	209863084	48675903	303227943
57163985	149728986	205830369	296225029	3023507	62510458	3023509	332215796	297671507	55620263
6831512	149632067								
**CCR4**									
1705894	297671782	332215473	109052678	296228310	194221518	62899791	301767336	154152187	1705895
26449155	225571128	291399774	290649642	126341582	149455250	327282179	326922159	118086158	224045511
**CCR6**									
2851567	332825448	332245368	297679621	74136427	296483830	301766648	73945797	194227505	291415344
8134362	61557091	126311276	149637480	166159172	326915616	224047748	327262258	301612736	AAVX01068499.1
153791315	213512406								
**CCR7**									
1352335	1352336	41054914	296202786	332847660	297701272	332258459	187475071	197210544	75070300
48374059	149724475	301779133	73965967	291406000	126308140	224086466	326934127	311771569	327275717
148222097	301626915	148922928	301616384	AAVX01326265.1	AAVX01024218.1				
**CCR8**									
1707884	71896604	326922147	296399392	224045515	3334152	27721715	10719948	114586090	297671676
332215595	296228403	149728750	57103782	301785880	303227947	291393287	126341586	224045501	124249288
AAVX01061874.1									
**CCR9**									
114152781	301623067	169145191	209155804	159155092	113951675	149632061	27229230	8134364	109041099
297671522	114586481	296225018	48675913	194221411	115311322	148356263	291393553	73985992	301754023
126341634	224045505	ENSACAP00000019440	AAVX01140752.1						
**CCR10**									
62298314	156104886	297701070	109115520	332260917	296201470	291406157	94536880	303227949	113205696
194216876	73965655	281344547	157819219	12643802	126307934	327275281			
**CXCR1 AND CXCR2**									
108936015	2494963	2494962	110825972	110825970	110825971	124357	157063152	2494966	194211303
194043812	149711459	57111007	6685568	301755776	301755774	296205556	23305862	297264881	1352454
125987816	2494967	2494968	547719	290542297	1352455	547718	126337864	126337862	290650152
2494965	81913011	326922912	78482916	71896165	327260352	327260354	148223850	149531934	292617830
3298340	185134540	47220980	118344614	189523763	3298358	47220226	AAVX01477245.1		
**CXCR3**									
2829400	222537776	332265855	297710303	149758513	311276475	75072906	75070299	281337759	291407679
75070286	76363509	76364160	185133155	213513980	47218519	169154030	58272233	58272235	301618339
49118568	327289267	169154032	185133520						
**CXCR4**									
46577576	3913205	3023448	114152796	75074809	3023451	75073173	46577575	75072692	128999
75072471	197253269	3023449	301784615	149730555	114149257	2494971	2494970	149637056	327260636
224056102	45382915	126326273	82241554	123884047	82249002	6318165	17223091	319099413	63102334
3551197	47215024	185133162	27802639						
**CXCR5**									
416718	311264026	291412974	73955058	301788472	291173052	416719	461630	297690401	332837885
332208422	126326932	326933405	71894759	301606664	326676225	ENSACAP00000016849	AAVX01026304.1		
**CXCR6**									
3121816	296225022	81917290	149018110	163915588	301607738	48675917	71153257	3121823	38503255
291173054	73985805	301754025	3121822	38503164	10719922	ENSMODP00000032629	ENSACAP00000013878	ENSOANP00000010838	
**CX3CR1**									
1351394	297671678	332215591	226342927	109041508	296228401	281352825	73990285	122136266	149729043
8134357	548703	238055160	126341584	149495131	224045513	296399391	326922149	50732904	327282177
**XCR1**									
1170008	114586489	332215787	297671509	109041073	296225024	194221407	303227953	48675911	73985988
301754029	291393557	12585214	157822209	126341638	149632065	326922097	113951667	224045503	327282173
292629502	66911140	326679306	301607740	291190313	225706150	47215603	AAVX01263959.1		

**Decoy receptors**									

**CCRL1**									
14285406	55621142	297671993	109049361	296228075	194221598	147898731	301781760	73990094	544463
291399807	68565247	109483837	301616697	148228890	317419986	292627507	47208340	148725584	ENSMODP00000032599
ENSOANP00000010818	ENSGALP00000019109	ENSACAP00000000733							
**CCRL2**									
114586515	108885280	297671505	75075026	296225035	48675905	115496362	194221403	73985969	281343590
108885281	157824077	291393561	126341646	149632075					
**CCBP2**									
20455469	114586376	297671602	296224947	291393242	149729016	57103810	301780460	194040849	62752046
14547935	14547939	126335978	224046888	50732143					
**CXCR7**									
115502380	55619711	297669797	109101586	296205948	149711234	132206	301789855	47117863	10720245
311273312	148356261	291414068	126314602	149633404	134085621	224054077	327260743	71896089	148223972
158254308	47226985	221307557	AAVX01259911.1						
**DARC**									
67476970	27734275	297663060	27734274	296229319	291397675	293341477	27734283	149755929	160332326
311254049	74006341	301783805	126307326	327287460					

**Viral receptors**									

**E1**									
124738385	124738389	124738361	9628003	124738365	124738377	124738369	124738381	124738383	124738379
124738375	124738373	124738371	124738391	124738393	124738399	124738395	124738401		
**ORF74**									
139472805	4154096	46519489	18653888	9626030	30348580	9631265	9629596	262285115	321496625
124738284	124738278	9628076	124738274						
**UL33**									
10998155	1717998	1717999	222354475	52139219	254770904	284518933	254771070	290564391	242345651
59803016	20026636	28412128	14251021	124248174	190886816	14916726	9845324	213159183	
**βHV**									
59800434	20026758	229270263	51556673	137159	20026757	229270249	51556669	229270262	51556671
229270250	33694234	229270261	23194507	229270231	51556668				
**pox**									
38229303	12085128	157939769	38229175	146746499	12084990	62637392	211956287	586239	211956433
62637539	226437997	226438057	2495049	226438017	13876663	226437981	226437989	226437993	

*The sequences obtained from the NCBI database are indicated by the GI numbers. The sequences with names starting with AAVX were obtained from the elephant shark genome project database, while the names stating with ENS indicate sequences obtained from the Ensembl database*.

### Amino acid sequence alignment and phylogenetic analysis

A multiple amino acid sequence alignment was produced with the alignment software MAFFT, version 6.857 (Katoh et al., 2002; Katoh and Toh, 2008). At first, 444 traditional CKRs were aligned. This result was manually refined, based on information about the secondary structures. Subsequently, 178 sequences consisting of decoy and viral receptors were added to the CKR alignment one by one, using the profile option of Clustal W (version 1.83; Thompson et al., 1994). Based on the alignment, an unrooted molecular phylogenetic tree was constructed by the neighbor-joining (NJ) method (Saitou and Nei, 1987). The genetic distance between every pair of aligned sequences was calculated as a maximum likelihood estimate (Felsenstein, 1996), under the JTT model (Jones et al., 1992) for the amino acid substitutions. The sites including gaps in the alignment were excluded from the calculation. The statistical significance of the NJ tree topology was evaluated by a bootstrap analysis (Felsenstein, 1985) with 1,000 iterative tree reconstructions. Two software packages, PHYLIP 3.5c (Felsenstein, 1993) and MOLPHY 2.3b3 (Adachi and Hasegawa, 1996), were used for the phylogenetic analyses. A cluster of decoy or viral receptors with a bootstrap probability greater than 80% was adopted as a group of receptors with different functions from the traditional CKRs.

### Calculation of the kullback–leibler information value

The multiple alignment thus obtained was reconstructed into 10 alignments, each consisting of two groups, the traditional CKRs and one of the decoy receptor groups or viral receptor groups. We then calculated the amino acid compositions of the two groups at each alignment site, according to the multiple alignment. We used the method adopted in PSI-BLAST (Altschul et al., 1997) to estimate the site-specific amino acid composition. The weighting method of Henikoff and Henikoff (1994) was used for the residue count. The weight for the pseudocount β, was set to 0.1. For the calculation of the pseudocount, λ*_u_*, a parameter for ungapped BLAST, was calculated for each alignment by the Newton–Raphson method (Ewens and Grant, 2001). When more than half of the sequences had gaps at an alignment site, the calculation of the site-specific amino acid composition and the following investigation were skipped. Next, the difference in the amino acid composition between two groups at each alignment site was calculated as the KL information value. The KL information value is defined as follows:

(1) $$\sum_{i=1}^{20}p(i)\log\frac{p(i)}{q(i)}$$

where *p* and *q* are the site-specific amino acid residue compositions for the two groups, which are estimated by the method described above. The parameter *i* indicates that the summation is obtained over 20 amino acid residues. KL information does not satisfy one of the distance axioms, symmetry. To satisfy this condition, the KL information was modified as follows:

(2) $$\sum_{i=1}^{20}p(i)\log\frac{p(i)}{q(i)}+\sum_{i=1}^{20}q(i)\log\frac{q(i)}{p(i)}$$

Formula 2 was used to predict the sites subjected to different functional constraints between the two groups. In this study, the functional constraint at a site of a protein sequence is defined as the extent of intolerance to mutation at the site, due to a reduction of the protein function by the mutation. This is a special case of the cumulative relative entropy developed by Hannenhalli and Russell (2000), which is applicable to an alignment consisting of multiple groups. When the KL information value of an alignment site was located in the top 5% of the distribution of KL information values for all of the sites, the site was regarded as a site under different constraints between the groups (Ichihara et al., 2004). Among them, the sites that fell in the gap region of CXCR4 in the alignment were neglected, because the subsequent analyses were performed based on mapping onto the CXCR4 structure.

### Statistical evaluation for bias in the spatial distribution of the sites under different constraints

**Figure 1:**
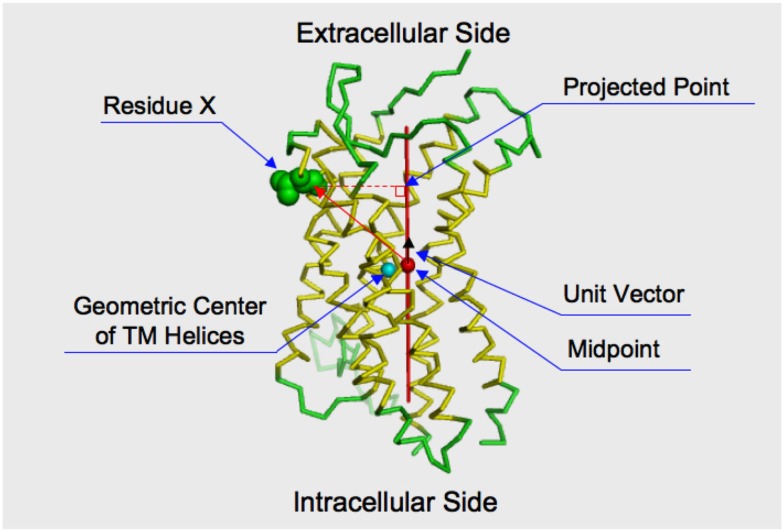
**Projection of a residue on the axis connecting the intracellular and extracellular sides of the receptor**. The structure of CXCR4 is shown by the ribbon model. The membrane spanning helices indicated by the structural element page for CXCR4 in GPCRDB (http://www.gpcr.org/7tm/) are colored yellow. The sphere colored cyan indicates the geometric center of the alpha carbons of the membrane spanning helices. The red axis connects the geometric center of extracellular loops and the N-terminal loop and that of the intracellular loops. The midpoint of the axis is indicated by a filled sphere colored red. The distance between the cyan and red spheres is close (3.26 Å). That is, the midpoint is considered to roughly reflect the geometric center of the transmembrane helices. How to take the orthogonal projection of an amino acid residue to the axis is shown by using Residue X. Consider a vector from the midpoint to the Cα atom of the residue. By taking an inner product between the vector and a unit vector, which runs along the axis and is originated from the midpoint. The projected point is obtained by taking the inner product.

We examined the statistical significance for the bias in the positions of the selected sites by the KL information values on the reference CXCR4 structure (PDB ID: 3ODU), using the following procedure. At first, we calculated the geometric center of the three extracellular loops (ECLs) and the N-terminal region, and that of the three ICLs. The coordinates of the Cα atoms were used for the calculation. The C-terminal region (residues 303–328) was not used in the calculation of the geometric center of the intracellular side, since this region extended into the cytosolic region. The chimeric lysozyme region was also neglected from the calculation. A unit vector on the axis connecting the two geometric centers, which originated from the midpoint between the geometric centers toward the geometric center of the extracellular side, was calculated. The inner product between the unit vector and a vector from the midpoint to the Cα atom of every residue, except for those of the chimeric lysozyme region, was then calculated. The inner product score indicated the projected position of the residue on the axis (see Figure 1). The positive or negative score corresponded to the extracellular or cytosolic location of a residue, respectively, relative to the geometric center. The distribution of the inner product scores for the residues selected by the KL information values was compared with those of the remaining residues by the two-sided *t*-test. The null hypothesis was the same for all of the tests: the average of the residues corresponding to the sites with large KL information values is the same as that of the remaining residues. For the statistical test, the function in the statistical computing software R, “*t*-test,” was used for the evaluation.

### Residue indication

The sites of each group selected by the KL information values are indicated on the corresponding sites of CXCR4 in this study. When the site has the number based on Ballesteros–Weinstein nomenclature (Ballesteros and Weinstein, 1995), the figure is also shown in the superscript. In this notation, the first digit indicates the number of the TM helix, and the following digit is the position counted from the most conserved site in each TM, to which the number 50 is assigned.

## Results

### The phylogenetic analysis

**Figure 2:**
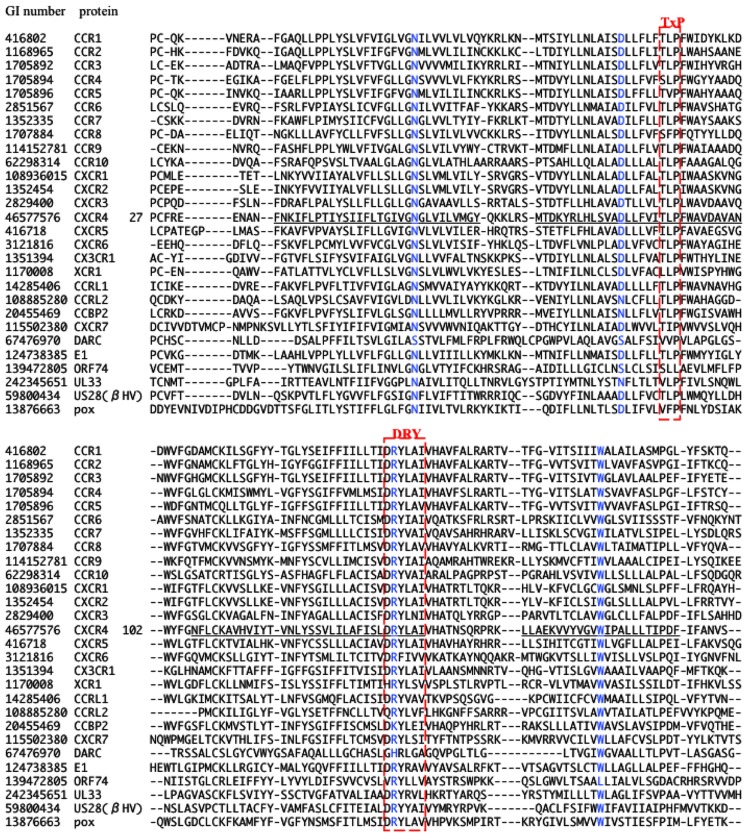

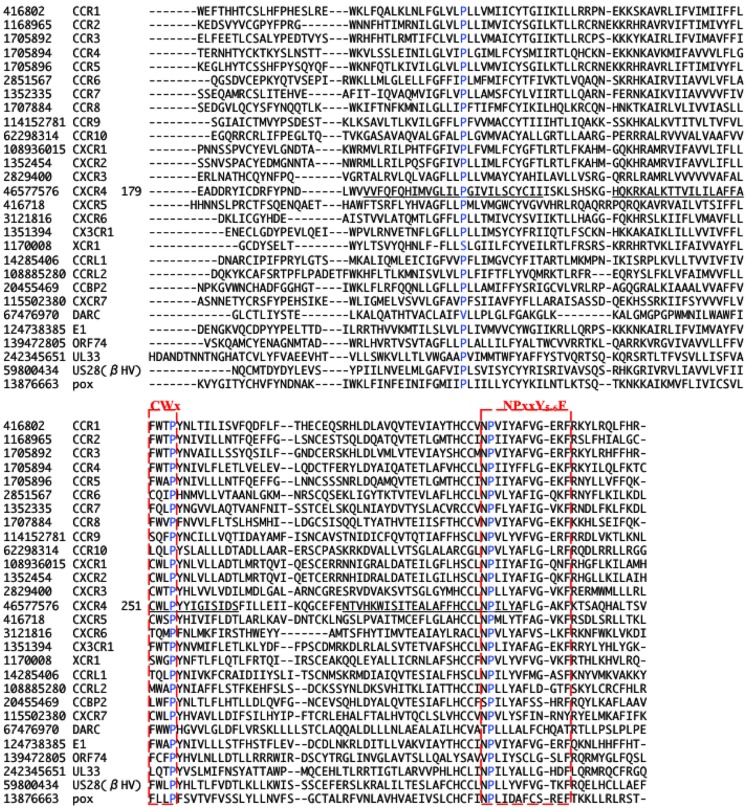
**Multiple amino acid sequence alignment of CKRs, decoy, and viral receptors**. The GI number and protein name of the representative protein from each group are shown at the left side of the aligned amino acid sequence. In the case of CXCR4, the residue number of the first residue of the aligned sequence is shown after the protein name, and the TM regions described in the GPCRDB (http://www.gpcr.org/7tm/) are indicated by underlines. The corresponding sites for x.50 of Ballesteros–Weinstein nomenclature are colored blue. Four motifs, TxP, DRY, CWxP, and NPxxY_5-6_F, are enclosed by red line.

**Figure 3:**
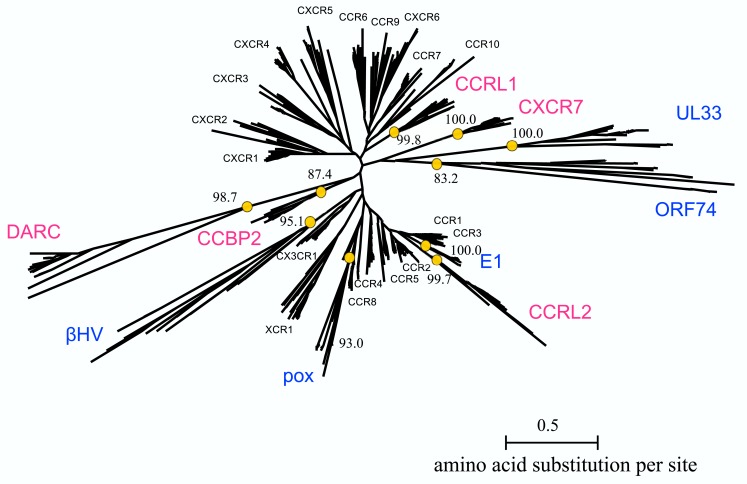
**Phylogenetic tree of CKRs and their homologs**. The tree for the 622 sequences listed in Table 1 is shown. The names of the CKRs (black), the decoy receptor groups (magenta), and the viral receptor groups (blue) are indicated near the receptor clusters. The bootstrap probabilities of the decoy and viral receptor groups are shown at the nodes corresponding to the common ancestors of the groups, which are indicated by circles.

The multiple alignment of 622 sequences were constructed, which is downloadable from the URL: http://seala.cbrc.jp/∼toh/suppl.html. The alignment of the representative sequences is shown in Figure 2. Based on the alignment, the phylogenetic tree of the CKRs and the decoy and viral receptors was constructed (Figure 3). Several clusters with high bootstrap probability (>80%) were identified in the tree, which included five decoy receptor groups and five viral receptor groups. The decoy receptor groups are referred to as CCRL1, CCRL2, CCBP2, CXCR7, and DARC, according to the constituent receptors. The numbers in each group were 23, 15, 15, 24, and 15, respectively. On the other hand, the viral receptor groups are referred to as E1, ORF74, UL33, βHV, and pox. The first three groups were named according to the constituent receptors. The βHV group consists US28, US27, and vGPCRs. Pox is a group of receptors derived from poxviruses. The numbers in each viral group were 18, 14, 19, 16, and 19. The evolutionary relationships between the CKRs and the decoy and viral receptors shown in the figure were roughly similar to those reported previously (Rosenkilde et al., 2001; Zlotnik et al., 2006). Murphy et al. (2000) suggest that the evolutionary rates of the CKRs are faster than those of the other GPCRs, because of the immune functions of CKRs. The long branch lengths suggested that the evolutionary rates of the receptors belonging to CCRL2, DARC, ORF74, UL33, βHV, and pox may be higher than those of the traditional CKRs, although we refrained from further examination of evolutionary rate accelerations in this study. In the subsequent analyses, each group of the decoy and viral receptors thus obtained was compared with the group of the traditional CKRs.

### Detection of sites with large KL information values

**Figure 4:**
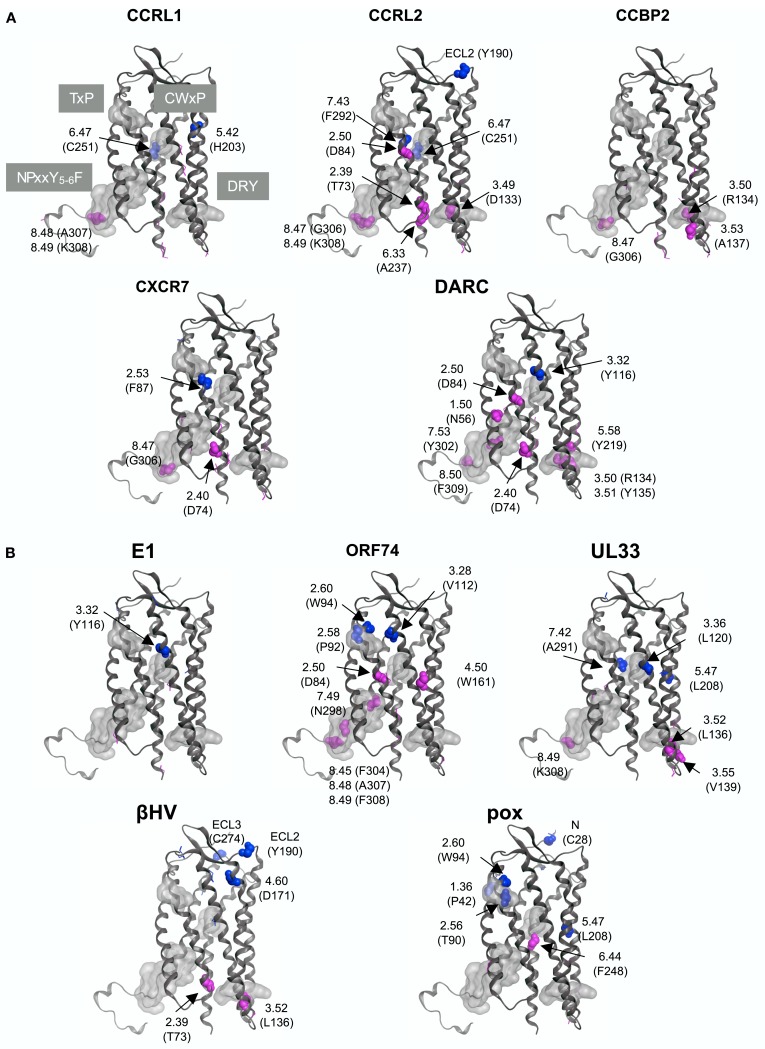
**Mapping of the sites with large KL information values on the CXCR4 structure**. The sites detected from the comparisons with **(A)** five decoy receptor groups and with **(B)** five viral receptor groups are mapped on the main chain structure of CXCR4. The residues corresponding to the detected sites with information about function and/or motif are depicted by space filling models, and are indicated according to the Ballesteros–Weinstein nomenclature. The corresponding amino acid residue types and numbers of CXCR4 are also shown in parentheses. On the other hand, the sites without any information are indicated by line models. The four motif regions are indicated by gray surface models. The residues that mapped on the extracellular side are colored blue, and those that mapped on the intracellular side are colored red.

The differences in the amino acid composition at each alignment site were examined between the traditional CKRs and each group of decoy and viral receptors. The sites with large KL information values in the top 5% are summarized in Table 2. The residues corresponding to such sites were mapped on the structure of CXCR4 (Figure 4). As shown in Table 2, about 11 ∼ 14 sites were selected from each group with the comparison of CKRs, and they included the sites in the sequences for GPCRs or the CKR-specific motif. Several sites that have been experimentally identified to be important for ligand binding or signaling were also selected. In addition, many uncharacterized sites were detected.

**Table 2:** **Selected sites with large KL information values**.

Residue (CXCR4)	Position	Region	B and W	Remarks	KL value	Frequency (%)				Reference
		
A. (Decoy receptors)						CKRs group			
**CCRL1**										
H203	Extra	TM5	5.42	Pocket	7.03					(L) Scholten et al. (2012)
						E	93.3			
C251	Extra	TM6	6.47	CWxP	6.71	C	39.7	F	31.7	(S) Nygaard et al. (2009)
						T	93.5			
A307	Intra	C	8.48	NPxxY_5-6_F	7.29	E	33.8	V	32.0	
						A	49.5			
K308	Intra	C	8.49	NPxxY_5-6_F	8.07	K	73.0			(L) Scholten et al. (2012)
						S	86.7		

Y121	Intra	TM3	3.37	Pocket	8.50	Y	67.0			
						V	40.3	S	34.3	
L125	Intra	TM3	3.41		7.19	L	55.6	F	38.0	
						Q	54.7			
T142	Intra	ICL2	3.58		6.67	T	33.2	V	32.2	
						P	59.7			
S144	Intra	ICL2	3.60		6.80	A	47.9			
						Q	36.5			
K230	Intra	ICL3	6.26		7.74	R	35.4			
						N	50.6			
G231	Intra	ICL3	6.27		8.26	N	39.9			
						I	43.4	W	31.8	
R235	Intra	ICL3	6.31		7.29	H	39.0			
						S	54.7			
T241*	Intra	ICL3	6.37		8.78	I	67.5			
						L	92.8	W	30.3	
I261	Extra	TM6	6.57		8.35	L	47.1			
						C	48.8			
L317	Intra	C	8.58		7.44	L	44.9			
						A	89.6			
**CCRL2**										
T73*	Intra	ICL1	2.39		9.69	T	91.9			(L) Scholten et al. (2012)
						E	38.7	G	34.7	
D84	Intra	TM2	2.50		8.20	D	95.4			(S) Rosenkilde et al. (2008)
						N	92.9			(S) Nygaard et al. (2010)
D133	Intra	ICL2	3.49	DRY	8.08	D	88.1			(L) Scholten et al. (2012)
						Q	84.4			
Y190	Extra	ECL2	–	Pocket	9.14	Y	47.5			(S) Zhou et al. (2001)
						R	47.1	K	33.4	
A237*	Intra	ICL3	6.33		9.72	A	79.1			(L) Scholten et al. (2012)
						L	83.5			
C251	Extra	TM6	6.47	CWxP	9.27	C	39.9	F	31.5	(S) Nygaard et al. (2009)
						M	72.8			
F292	Extra	TM7	7.43		10.56	F	46.3			(L) Scholten et al. (2012)
						T	61.6			(L) Choi et al. (2005)
G306	Intra	C	8.47	NPxxY_5-6_F	11.94	G	96.0			
						D	93.1			
K308	Intra	C	8.49	NPxxY_5-6_F	8.96	K	73.8			(L) Scholten et al. (2012)
						T	36.3			

E31	Extra	N	–	Pocket	9.72					
						Y	78.0			
A141	Intra	ICL2	3.57		8.63	A	82.2			
										
I215	Intra	TM5	5.54		8.31	M	72.6			
						F	69.4			
I223	Intra	ICL3	5.62		8.16	I	40.0	L	31.8	
						R	66.8			
**CCBP2**										
R134	Intra	ICL2	3.50	DRY	10.75	R	99.0			(S) Deupi and Standfuss (2011)
						K	65.9			(S) Holst et al. (2010)
A137	Intra	ICL2	3.53	DRY	9.22	A	77.1			
						E	53.4			
G306	Intra	C	8.47	NPxxY_5-6_F	9.35	G	96.2			
						S	53.7		

V59	Intra	TM1	1.53		7.61	V	94.3			
						L	81.3			
T142	Intra	ICL2	3.58		10.12	T	33.2	V	31.9	
						Q	57.0			
S144	Intra	ICL2	3.60		7.54	A	48.0			
						H	33.5			
A152	Intra	ICL2	4.41		7.65					
						K	60.6			
Y157	Intra	TM4	4.46		7.47	C	49.1	S	36.1	
										
S224	Intra	ICL3	5.63		7.68					
						C	80.7			
K230	Intra	ICL3	6.26		7.89	R	36.1	Q	34.4	
						L	67.8			
Q233	Intra	ICL3	6.29		11.12	K	36.1			
						G	91.9			
**CXCR7**										
D74	Intra	ICL1	2.40		11.53	D	72.0			(L) Scholten et al. (2012)
						H	90.8			
F87	Extra	TM2	2.53		10.25	F	75.4			(L) Tian et al. (2005)
						V	84.5			
G306	Intra	C	8.47	NPxxY_5-6_F	11.52	G	96.2			
						N	66.8		

K38	Extra	N	1.32	Pocket	10.59					
						Y	86.7			
G55	Intra	TM1	1.49		11.17	G	97.8			
						A	91.1			
M63	Intra	TM1	1.57		10.87	L	49.7			
						N	90.6			
M72*	Intra	ICL1	2.38		10.57	M	41.0			
						E	54.2	D	37.3	
L86	Extra	TM2	2.52		9.89	L	79.1			
						C	55.5	W	38.4	
A141	Intra	ICL2	3.57		11.00	A	81.9			
						F	67.4			
C218*	Intra	ICL3	5.57		10.28	C	94.0	R	41.7	
						F	79.9			
K236	Intra	ICL3	6.32		10.01	K	56.4			
						S	54.7			
L238*	Intra	ICL3	6.34		10.15	V	32.9	I	31.4	
						R	60.5			
L244	Intra	TM6	6.40		11.42	V	54.0			
						Y	92.6			
K271	Extra	ECL3	–		10.02					
						F	79.8			
**DARC**										
N56	Intra	TM1	1.50		11.51	N	98.8			(S) Rosenkilde et al. (2008)
						S	78.2			(S) Nygaard et al. (2010)
D74	Intra	ICL1	2.40		11.91	D	72.3			(L) Scholten et al. (2012)
						R	44.9	W	30.1	
D84	Intra	TM2	2.50		11.61	D	95.5			(S) Rosenkilde et al. (2008)
						S	73.8			(S) Nygaard et al. (2010)
Y116	Extra	TM3	3.32	Pocket	11.54	Y	57.9			(L) Scholten et al. (2012)
						W	63.8			(L) Surgand et al. (2006)
R134	Intra	ICL2	3.50	DRY	12.36	R	98.9			(S) Deupi and Standfuss (2011)
						G	61.7			(S) Holst et al. (2010)
Y135	Intra	ICL2	3.51	DRY	13.42	Y	92.6			
						P	83.1			
Y219*	Intra	ICL3	5.58		12.72	Y	96.2			(S) Holst et al. (2010)
	G	52.1			
Y302	Intra	TM7	7.53	NPxxY_5-6_F	11.86	Y	95.7			(L) Scholten et al. (2012)
						L	75.4			(S) Rosenkilde et al. (2008)
F309	Intra	C	8.50	NPxxY_5-6_F	12.49	F	98.7			(S) Rosenkilde et al. (2008)
						A	54.7		

V214	Intra	TM5	5.53		11.34	V	52.4			
						P	93.5			
C218*	Intra	ICL3	5.57		12.38	C	94.1			
						L	63.6			
T241*	Intra	ICL3	6.37		13.28	I	67.3			
						W	75.7			
L246	Intra	TM6	6.42		12.77					
						W	96.0			
C296	Intra	TM7	7.47		11.56	C	88.7			
						V	64.4			

**B. (Viral receptors)**										

**E1**										
Y116	Extra	TM3	3.32	Pocket	7.62	Y	56.1			(L) Scholten et al. (2012)
						C	88.6			(L) Surgand et al. (2006)
Q66	Intra	ICL1	1.60		6.34					
						M	83.5			
A95	Extra	TM2	2.61		8.18	A	66.0			
						M	63.8			
V99	Extra	TM2	2.65		6.66	A	34.8			
						G	84.8			
N106	Extra	ECL1	3.22		6.48					
						I	71.1			
S123	Intra	TM3	3.39	Pocket	8.02	G	49.6	S	36.4	
						Q	54.8			
G207	Extra	TM5	5.46	Pocket	7.77	G	90.7			
						S	84.3			
C220	Intra	ICL3	5.59		6.62					
						Y	47.3	W	41.2	
G231	Intra	ICL3	6.27		9.44	N	39.6			
						P	84.4			
**ORF74**										
D84	Intra	TM2	2.50		11.25	D	95.6			(S) Rosenkilde et al. (2008)
						S	63.3			(S) Nygaard et al. (2010)
P92	Extra	TM2	2.58	TxP	11.63	P	98.4			(L) Govaerts et al. (2001)
						L	59.7			(S) Wu et al. (2010)
W94	Extra	TM2	2.60	Pocket	9.67	W	74.7			(L) Scholten et al. (2012)
										(S) Rosenkilde et al. (2010)
V112	Extra	TM3	3.28	Pocket	9.31	V	37.3			(L) Scholten et al. (2012)
						E	38.4			
W161	Intra	TM4	4.50			W	99.3			(C) Ballesteros and Weinstein (1995)
						F	32.9			
N298	Intra	TM7	7.49	NPxxY_5-6_F	11.57	N	94.9			(S) Rosenkilde et al. (2008)
						V	38.2			(S) Nygaard et al. (2010)
F304	Intra	C	8.45	NPxxY_5-6_F	9.17	F	94.6			
						L	57.4			
A307	Intra	C	8.48	NPxxY_5-6_F	10.63	E	33.4	V	32.2	
						S	94.5			
K308	Intra	C	8.49	NPxxY_5-6_F		K	73.8			(L) Scholten et al. (2012)
						L	48.0		

Y76 *	Intra	ICL1	2.42		8.84	Y	60.6	F	32.7	
						L	81.9			
A83	Intra	m	2.49		10.43	A	55.3	S	40.1	
						N	62.9			
H140 *	Intra	ICL2	3.56		9.90	H	49.5			
						F	50.0			
A237 *	Intra	ICL3	6.33		9.31	A	79.4			
						V	65.4	I	30.2	
**UL33**										
L120	Extra	TM3	3.36	Pocket	13.59	F	70.9			(L) Surgand et al. (2006)
						C	96.4			
L136	Intra	ICL2	3.52	DRY	11.63	L	66.3			
						R	74.7			
V139	Intra	ICL2	3.55	DRY	12.66	V	90.5			
						H	74.4			
L208	Extra	TM5	5.47	Pocket	13.48	F	70.1			(S) Holst et al. (2010)
						G	95.2			
A291	Extra	TM7	7.42		11.64	A	60.0	G	31.0	(L) Scholten et al. (2012)
						P	95.1			
K308	Intra	C	8.49	NPxxY_5-6_F	11.86	K	73.3			(L) Scholten et al. (2012)
						D	74.3		

G55	Intra	TM1	1.49		12.83	G	97.9			
						L	46.4	M	37.0	
W102	Extra	ECL1		Pocket	12.73	W	96.3			
						L	34.3			
A141	Intra	ICL2	3.57		11.49	A	82.0			
						R	83.8			
G207	Extra	TM5	5.46	Pocket	15.89	G	90.4			
						W	96.7			
C218 *	Intra	ICL3	5.57		11.64	C	93.6			
						F	96.2			
I222 *	Intra	ICL3	5.61		11.47	I	75.4			
						F	96.2			
Y256	Extra	TM6	6.52	Pocket	11.30	N	77.3			
						V	48.6			
C296	Intra	TM7	7.47		12.72	C	88.6			
						L	59.0			
**βHV**										
T73 *	Intra	ICL1	2.39		9.04	T	92.5			(L) Scholten et al. (2012)
						S	50.1			
L136	Intra	ICL2	3.52	DRY	10.76	L	66.1			
						S	32.8			
D171	Extra	TM4	4.60	Pocket	8.92					(L) Tian et al. (2005)
						Y	47.7			
Y190	Extra	ECL2	–	Pocket	11.26	Y	47.3			(S) Zhou et al. (2001)
						N	70.6			
C274	Extra	ECL3	–		12.37	C	96.2			(C) Wu et al. (2010)

W102	Extra	ECL1	–	Pocket	11.59	W	96.5			
F104	Extra	ECL1	–		10.98	F	81.4			
						S	31.9			
K110	Extra	ECL1	3.26		10.19	K	85.5			
						I	44.8			
N119	Extra	TM3	3.35		8.46	N	48.1	G	33.1	
						P	37.0			
H140 *	Intra	ICL2	3.56		8.66	H	49.0			
						W	38.6			
W283	Extra	TM7	7.34		9.65	A	76.5			
						F	37.0			
**pox**										
C28	Extra	N	–		9.87	C	90.9			(C) Wu et al. (2010)
						Y	37.6			
P42	Extra	TM1	1.36		7.91	P	70.4			(L) Scholten et al. (2012)
						I	62.2			
T90	Extra	TM2	2.56	TxP	6.79	T	68.6			(S) Govaerts et al. (2001)
										(S) Alvarez Arias et al. (2003)
W94	Extra	TM2	2.60	Pocket	10.13	W	74.6			(L) Scholten et al. (2012)
						I	39.4			(S) Rosenkilde et al. (2010)
L208	Extra	TM5	5.47	Pocket	8.06	F	70.8			(S) Holst et al. (2010)
						M	67.6			
F248	Intra	TM6	6.44	Pocket	9.82	F	83.3			(S) Deupi and Standfuss (2011)
						S	49.9	T	33.8	(L) Surgand et al. (2006)

P27	Extra	N	–		7.38	P	56.2			
						D	46.0	E	30.8	
E31	Extra	N	–	Pocket	11.70					
						Y	95.2			
F36	Extra	N	1.30		7.00	F	64.3			
						V	35.1			
L61	Intra	TM1	1.55		6.93	L	41.2			
						T	44.2			
I215	Intra	TM5	5.54		7.28	M	73.4			
						L	65.3			
I221 *	Intra	ICL3	5.60		6.16					
						K	85.4			
S227	Intra	ICL3	–		6.53	L	45.4			
						K	85.4			
E277	Extra	ECL3	7.28		6.16	S	33.3			
						L	33.8	F	30.7	

(A) The list of the sites detected from the comparisons with five decoy receptor groups. (B) The list of the sites detected from the comparisons with five viral receptors. Each row corresponds to a site with a large KL information value. The first column indicates the residue type and the residue number of CXCR4, to which the selected site corresponded. “*” Indicates a site located within 5 Å from the DRY motif in the CXCR4 structure. The second column indicates whether the site is located on the extracellular or intracellular side. The location was determined for the *t*-test (see Materials and Methods). The third column shows the position of the site in the primary structure of a GPCR (N-terminus, TM, ICL, ECL, and C-terminus). The fourth column indicates the site by the Ballesteros–Weinstein nomenclature. The fifth column provides remarks about the site, such as experiments and motifs. The “pocket” in this column was calculated at the CASTp site (Liang et al., 1998). A blank entry in the fifth column means that the site has not been characterized yet. The sixth column indicates the KL value of each site. The seventh column indicates the frequencies of the residues at each site. The upper half of the column indicates the frequencies for CKRs, and the lower half indicates the frequencies for the group under comparison. Only the residues with frequencies greater than 30% are shown. The eighth column indicates whether the site is involved in ligand binding (L), signaling (S), or conservation (C). The literature for experimental evidence or observations of the characteristics is also shown in the column, although the experiments were not always performed with the receptors under consideration, but with the homologs in the CKR family.

The DRY (Asp-Arg-Tyr) motif of the GPCRs is conserved as the sequence DRYLAIV in the traditional CKRs, from the end of TM3 to ICL2 (Graham, 2009). The motif is related to signal transduction, through interactions with G proteins. The conserved R134^3.50^ is involved in the interchanges between the inactive and active conformations of GPCRs. In the inactive conformation, this Arg interacts with its neighboring residue, D133^3.49^, but in the active conformation, the residue interacts with Y219^5.58^ (Holst et al., 2010). The sites in the DRY region of the DRYLAIV sequence were only detected from the analyses with the decoy receptor groups, CCRL2, CCBP2, and DARC. In addition, Y219^5.58^ was also detected from the analysis with the DARC group. On the other hand, the sites in the LAIV^3.52 ∼ 3.55^ region of the DRYLAIV sequence were detected from the examinations with the decoy and viral receptor groups, CCBP2, UL33, and βHV. The CWxP motif is located in the middle of TM6. This W252^6.48^ is believed to function as a micro-switch in the receptor activation mechanism, and P254^6.50^ creates a kink in this helix, around which TM7 performs its rigid body movements during activation (Nygaard et al., 2009). The corresponding sites of this motif were detected from the analyses of two decoy receptor groups, CCRL1 and CCRL2, but not from those of any viral receptor group. The fifth site of the NPxxY_5-6_F motif in TM7, Y302^7.53^ functions in the interchange of an inactive rotamer conformation (Nygaard et al., 2009). The sites of this motif were detected from the investigations with every decoy receptor group and two viral receptor groups, ORF74 and UL33. The TxP motif of TM2 is known as a specific motif of the traditional CKRs. It is known that the TxP motif in TM2 is specific for traditional CKRs (Govaerts et al., 2001). The third site of the TxP motif, P92^2.58^, bends the helix, which determines the intra-helical location that is involved in the receptor activation. The sites of the motif were detected from the analyses of two viral receptor groups, the ORF74 and pox groups, but not from the assessment with any decoy receptor group. In addition, several sites corresponding to highly conserved positions in GPCRs, which are denoted as x.50 by the Ballesteros–Weinstein nomenclature, such as N56^1.50^ and D84^2.50^, were detected from analyses of several groups (see Table 2). Table 2 also shows the other important residues experimentally identified as having binding or signaling functions.

We examined which sites were commonly selected from the comparisons. No site was shared in all of the comparisons. Furthermore, there was no site commonly detected from the analyses with all of the decoy receptor groups or all of the viral receptor groups. However, several sites were detected from the different comparisons. For example, the sites corresponding to D74^2.40^, D84^2.50^, R134^2.50^, A141^3.57^, T142^3.58^, S144^3.60^, C218^5.57^, K230^6.26^, T241^6.37^, C251^6.47^, G306^8.47^, and K308^8.49^ were detected from at least two assessments with decoy receptor groups. Most of these sites are located in ICL2, 3, and the C-terminal region. Among these sites, D84^2.50^, A141^3.57^, C218^5.57^, and K308^8.49^ were also detected from at least one analysis with the viral receptor group. W94^2.60^, W102 (ECL1), L136^3.52^, H140^3.56^, G207^5.46^, L208^5.47^, and K308^8.49^ were detected from at least two analyses of the viral receptor groups. None of them, except for K308^8.49^, was detected from the analyses of any decoy receptor group.

### Statistical test for the spatial bias of the sites with large KL information values

**Figure 5:**
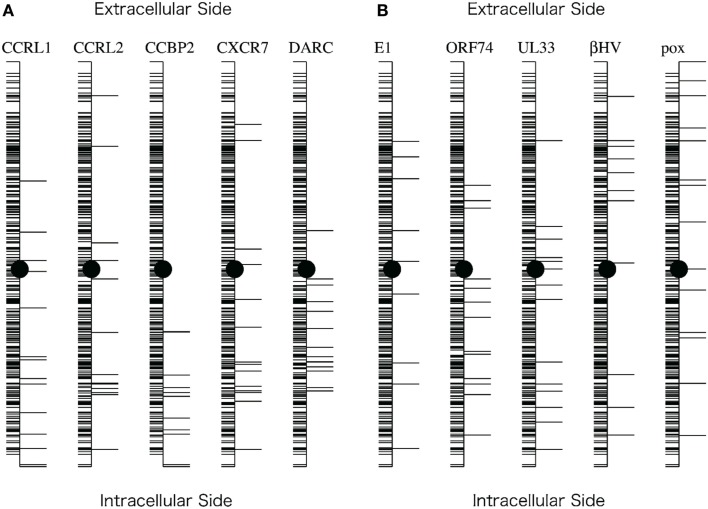
**Projection of the residues on the axis connecting the intracellular and extracellular sides of the receptor**. **(A)** Projections corresponding to the five comparisons with the decoy receptor groups. **(B)** Projections corresponding to the five comparisons with the viral receptor groups. The axis generation method and the projection method are described in the Section “Materials and Methods.” In each figure, the vertical line indicates the axis. Horizontal lines on the right side of the axis indicate the projected positions of the residues corresponding to the sites selected based on the KL information values. Horizontal lines on the left side indicate the projected positions of the remaining residues. The filled circles indicate the midpoint of the axis.

As shown in Figure 4, the distribution of the sites selected from the analyses with the decoy receptor groups seemed to be biased toward the cytosolic side of the CKR structure. In contrast, there did not seem to be any trends in the distribution of the sites obtained from the analyses with the viral receptor groups. To quantitatively examine the observations, the residues corresponding to the selected sites and the remaining residues were projected on the axis connecting the center of gravity of the ECLs including the N-terminal region, and that of the ICLs (see Figure 5). Based on the projection on the axis, *t*-tests were performed as described in the Section “Materials and Methods.”

The results of the *t*-tests are summarized in Table 3. As shown in this table, the null hypothesis was rejected in three cases of the analyses with decoy receptor groups, CCRL1, CCBP2, and DARC, under the significance level of 5%. To examine the bias further, the one-sided *t*-test was applied to the observations about the decoy receptor groups. The null hypothesis was the same as that of the two-sided test, but the alternative hypothesis was that the average of the residue with the large KL value is smaller than that of the remaining residues. We found that the null hypothesis was rejected for four cases with decoy receptor groups, CCRL1, CCBP2, DARC, and CXCR7 (data not shown). That is, the distribution of the residues corresponding to the sites with large KL information values of the decoy receptor groups, except for CCRL2, was biased toward the intracellular side of the receptor. The two-sided *t*-test was also applied to the analyses of the viral receptor groups. In all cases, the null hypothesis was not rejected. This result suggested that the residues selected by the KL information values of the viral receptors were distributed on both the extracellular and intracellular sides.

**Table 3:** **Results of *t*-tests**.

**DECOY RECEPTORS**	
CCRL1	3.87 × 10^−3^
CCRL2	0.142
CCBP2	3.37 × 10^−7^
CXCR7	0.066
DARC	1.51 × 10^−4^
**VIRAL RECEPTORS**	
E1	0.981
ORF74	0.080
UL33	0.098
βHV	0.308
pox	0.144

The *p*-value for each two-sided *t*-test is shown. The details of the tests are described in the Section “Materials and Methods.”

## Discussion

### Decoy receptors

The difference in the amino acid composition at an alignment site between two receptor groups, as evaluated by the KL information value, was considered to reflect the difference in the functional constraints at the site between the groups. As described above, decoy receptors are able to bind to CKs, but do not induce signaling. The sites detected by the KL information value would reflect the functional difference. Actually, the sites included in several motifs, such as DRY, CWxP, and NPxxY_5-6_F, which are involved in signaling, were detected. The bias in the locations of the detected sites on the intracellular side was statistically significant by the two-sided or one-sided *t*-test in four out of five decoy receptor groups. Especially, all of the sites detected from the analysis of CCBP2 were located on the intracellular side. The test with the CCRL2 group was the only one that did not suggest a statistically significant bias in the distribution of the detected sites. As described above, CCRL2 is also able to bind to chemerin (Yoshimura and Oppenheim, 2011). The adaptation to the new ligand may have introduced the change in the functional constraints on the extracellular side, which may be the reason why the null hypothesis was not rejected. This observation suggested that the functional divergence of CCRL2 was induced under different selective pressure, as compared to the other decoy receptors after gene duplication. CCRL2 forms a gene cluster together with the genes for CCR1, 2, 3, and 5 in several mammalian genomes (Nomiyama et al., 2011). The close relationship of CCRL2 to these CKRs and its distant relationship to the other decoy receptors in the phylogenetic tree (Figure 3) were consistent with the conservation of the gene orders in the genomes, although the bootstrap probabilities for the relationships were not always high. The evolutionary relationship and the conserved gene order, together with the acquisition of binding activity to a new ligand, suggested a unique evolutionary position of CCRL2 relative to the other decoy receptors.

The lack of signal transduction activity in the decoy receptors is attributed to the degeneration of the DRY motif (Comerford et al., 2007). Our study suggested that the degenerations of other motifs and functional residues may also be related to functional changes. For example, two decoy groups, CCRL1 and CXCR7, contained the typical DRY motif. However, the sites in other motifs that are related to the conformational change associated with the active-inactive switch had large KL information values in these decoy receptors. This observation suggested that the constraints for the residue conservation at the sites in the traditional CKRs are looser in the two decoy receptor groups (see Table 2). In addition to the motif sites, the highly conserved sites in the TM regions of GPCRs, including the traditional CKRs (x.50 in the Ballesteros–Weinstein nomenclature), had large KL information values in the analyses with several decoy receptor groups. The use of different amino acid residues at such sites may lead to functional and/or structural changes. Several sites with uncharacterized functional relationships also showed large KL information values. Most of them were found in ICLs 2 and 3. As these loops are considered to interact with G proteins, the sites detected on the loops may be involved in the loss of the signaling function of the decoy receptors.

### Viral receptors

We anticipated that the sites detected from the analyses with the viral receptor groups would be found on the extracellular side, since viral receptors exhibit signaling activity without ligand binding. As described above, however, the sites with the large KL information values were found not only on the extracellular side, but also on the intracellular side. We examined the detected sites from the different viewpoint. CASTp^4^ (Liang et al., 1998) is a program to identify pocket regions in a given tertiary structure. When we applied CASTp to the coordinates of CXCR4, the pocket region corresponding to the ligand binding cavities of GPCRs was reported with the highest score. The residues consisting of the pocket region were mainly projected on the extracellular side of the axis (see Figure 1), although some residues were projected on the intracellular side. The numbers of detected sites located in the pocket regions of the five decoy receptor groups were 2, 2, 0, 1, and 1, whereas 3, 2, 5, 3, and 4 sites were located in the pocket regions of the five viral receptor groups (see Table 2). The number of sites was transformed into the ratio to the total number of detected sites for each receptor group. The one-sided *t*-test showed that the difference in the ratios between the decoy and viral receptor groups was statistically significant (*p*-value = 0.003864). That is, more sites were detected in the pocket region in the viral receptor groups, as compared to the decoy receptor groups. As shown in Table 2, in addition, about half of the sites in the pocket region have been characterized as being involved in ligand recognition. These sites are often occupied by conserved, bulky amino acid residues in CKRs. The result suggested that the functional constraints at the ligand binding region are different between the viral receptors and the traditional CKRs, as we first expected.

The sites in the DRY motif were not detected in any of the viral receptor groups. This motif was basically conserved in the viral receptors, except for the ORF74 group. A previous study reported that ORF74 performs signal transduction, despite the fact that the DRY motif is changed to DTW (Rosenkilde et al., 2005). They also showed that the introduction of the DRY sequence into ORF74 induces functional reduction. In our study, the sequences collected as the ORF74 group showed variations in this region. For example, equid herpesvirus 2 has DTW, whereas the rodent and primate herpesviruses have xRC or xRY. Each variation includes the residues identical to those of the original DRY motif, which may have reduced the KL information value and led to the failure in the detection of the sites. Instead, the sites in the TxP and NPxxY_5-6_F motifs and the sites spatially surrounding the DRY motif were detected from the analysis of the ORF74 group (see Table 2). The amino acid replacements in the two motifs, which are considered to be involved in the conformational change, and those of the residues near the DRY motif may have contributed to the maintenance of the signaling activity of ORF74, despite the deviation from the typical DRY motif. In contrast, no sites in any motif were detected from the comparison with the E1 group. The E1 receptor reportedly lacks constitutive signaling activity (Rosenkilde et al., 2008). The conservation of the motifs suggested the difference in the signaling functions between the E1 group and other viral receptor groups.

We had not expected to detect the sites on the intracellular side from the comparisons with the viral receptor groups, since these receptors exhibit signaling activity without ligand binding. However, quite a few sites with large KL information values were also found on the intracellular side. As described above, the overlap of the selected sites between the decoy receptors and the viral receptors was small. The difference in the selected sites on the intracellular side between the viral receptor groups and the decoy receptor groups may be basically related to the difference in the activities of the receptor groups. That is, the sites of the viral receptor groups under the constraint to maintain the signaling without ligand binding may be different from the sites of the decoy receptor groups, where the functional constraints may have been weakened due to the loss of the signaling activity.

## Conclusion

We have identified the alignment sites (and the corresponding amino acid residues) that may be responsible for the functional changes from CKRs to decoy receptors or viral receptors. The distributions of the identified residues on the tertiary structure seemed to reflect the functional differences. This prediction could be examined by an experimental study, such as amino acid replacement, or a computational study with molecular dynamic simulations. Such studies could provide deep insights into the mechanism of GPCR signaling through conformational changes. The experimental and computational confirmations of our prediction remain as future endeavors.

## Conflict of Interest Statement

The authors declare that the research was conducted in the absence of any commercial or financial relationships that could be construed as a potential conflict of interest.

## Footnotes

^1^http://www.ncbi.nlm.nih.gov/BLAST/

^2^http://www.ensembl.org/index.html

^3^http://esharkgenome.imcb.a-star.edu.sg/

^4^http://sts.bioengr.uic.edu/castp/
